# Are Children Most of the Submerged Part of SARS-CoV-2 Iceberg?

**DOI:** 10.3389/fped.2020.00213

**Published:** 2020-04-30

**Authors:** Stefano Passanisi, Fortunato Lombardo, Giuseppina Salzano, Giovanni Battista Pajno

**Affiliations:** Department of Human Pathology in Adult and Developmental Age Gaetano Barresi, University of Messina, Messina, Italy

**Keywords:** children, coronavirus, COVID-19, Italy, outbreak, pandemic, transmission

## Introduction

Since the end of 2019, a novel coronavirus disease 2019 (COVID-19) caused by a novel coronavirus, severe acute respiratory syndrome coronavirus 2 (SARS-CoV-2) has been developing in Wuhan, Hubei Province, China (1). In very few months it has spread all over the world and the World Health Organization on March 11, 2020 declared COVID-19 a pandemic.

Italy is currently one of the most affected countries in Europe. At the time of this publication, 120,290 cases of SARS-CoV-2 infection have been detected. Median age of infected patients is 62 years. Interestingly, only 1.5% of patients diagnosed with SARS-CoV-2 are aged 0–18 years. Thus far, among the 14,381 subjects who died with SARS-CoV-2 in Italy, there was only one child (2). These data are in concordance with those reported from other European countries and a recent review of 72,314 COVID-19 patients by the Chinese Center for Disease Control and Prevention that revealed that there were <1% of pediatric cases. Furthermore, among children younger than 10 years of age no deaths were reported (3). Even during the outbreak of severe acute respiratory syndrome coronavirus (SARS-CoV) and middle-east respiratory syndrome coronavirus (MERS-CoV) the rate of infected children was quite low (5–7% and 2–3%, respectively) (4, 5).

## SARS-CoV-2 Infection in Children

The most common symptoms in children with confirmed SARS-CoV-2 are fever, dry cough, pharyngeal erythema, and fatigue (6). Recently, Dong et al. described a cohort of 2,143 Chinese children with suspected or confirmed COVID-19. The authors reported that among symptomatic children, only 5% had dyspnoea or hypoxemia and 0.6% presented acute respiratory distress syndrome or multiorgan failure (7). Early laboratory markers that are typically associated to SARS-CoV-2 infection in adults such as lymphopenia, increased liver enzymes, anemia, increased inflammatory markers, are very rare in children (8).

The reasons why children appear to be less affected and if infected develop milder clinical pictures due to COVID-19 are still uncertain. Several hypothesis have been put forward. Children may have a more effective response against SARS-CoV-2 as their immune system may be strengthened by the recurrence of several viral infections toward which they tend in the first years of life. In addition, adults seem to have a more vigorous immune response that may lead to “cytokine storm” which will further deteriorate lung injury (9). This theory is supported by the rare increase of inflammatory markers in children with confirmed COVID-19, as reported above. Another interesting hypothesis is related to angiotensin-converting enzyme 2 (ACE2). This enzyme is widely expressed in organs, including lungs in which it seems to act in a protective manner especially against severe respiratory virus disease (10). The extracellular domain of ACE2 has been demonstrated to be the functional receptor for the spike protein of SARS-CoV, and recently, of the SARS-CoV-2 (11). Experiments in rats show that the pulmonary renin angiotensin system undergoes an age-dependent imbalance toward a more pronounced inflammation and more severe lung injury (12). In fact, ACE2 expression was seen to significantly decrease with increasing age in rat lung models, with old male rats having the lowest levels (13). Therefore, a higher concentration of ACE2 in lung pneumocytes in children could explain the rarity of severe clinical manifestations due to SARS-CoV-2 infection. Other suggested reasons that may contribute include lack of smoking, less exposure to air pollution and fewer underlying chronic conditions in children rather than adults (14). However, further studies are awaited to better investigate the underlying mechanisms that lead to milder disease presentation in the pediatric population.

## Discussion

Although more than a million people have already had a confirmed diagnosis of SARS-CoV-2 worldwide, there is a strong impression that the true prevalence of SARS-CoV-2 infection is much higher. For instance, one study suggested that 86% of all early SARS-CoV-2 infections in China remained undiagnosed (15). Pharyngeal and nasal swabs, which represent the most used diagnostic investigations, are mostly reserved to those individuals who present severe illness. In addition, the sensitivity of these tests is not yet known, but numerous false negatives may occur, since the virus may have translocated from the upper to the lower airways. Therefore, people with confirmed diagnosis may only be the tip of the iceberg of SARS-CoV-2 pandemic. The majority of infected children do not undergo diagnostic investigations for COVID-19 as they are asymptomatic or mildly symptomatic. Available data indicate that children who become infected with SARS-CoV-2 may have more upper respiratory tract rather than lower respiratory tract involvement (7). This may explain the low rate of infected pediatric patients reported in epidemiological studies. Fecal shedding persists in the stool for several weeks after diagnosis (16). Extended shedding in nasal secretions and stool may have remarkable implications for community spread in kindergartens, schools, and at home (17). Therefore, the role of children in community-based viral transmission should be carefully investigated to understand how much it can actually affect public health.

In the meantime, several countries have issued strict governmental decrees prohibiting movement in public places except for justifiable work reasons, basic necessities (i.e., food shopping), and health emergencies. School closures were among the first measures which had been adopted. According to UNESCO monitoring, over 160 countries have implemented nationwide closures, impacting over 87% of world's student population. Together these hard interventions seem to give encouraging results (18): reducing the contagion among the pediatric population could be a first step to curb the spread of COVID-19.

## Author Contributions

SP drafted and wrote the paper. GS and FL contributed to the discussion. GP reviewed and approved the paper.

## Conflict of Interest

The authors declare that the research was conducted in the absence of any commercial or financial relationships that could be construed as a potential conflict of interest.
